# Silver Nanoparticles Suppress Apple Fire Blight: Antibacterial Activity, Reduced Carbohydrate-Containing EPS Content, and Altered Host Antioxidant Responses

**DOI:** 10.3390/plants15203208

**Published:** 2026-10-19

**Authors:** Alexander G. Khina, Maria T. Mukhina, Alexander S. Gordeev, Liliya R. Biktasheva, Dmitry M. Mikhaylov, Georgii V. Lisichkin, Yurii A. Krutyakov

**Affiliations:** 1 Department of Chemistry, M. V. Lomonosov Moscow State University, Moscow 119991, Russia; lisich@petrol.chem.msu.ru; 2 NTI Center “Digital Materials Science: New Materials and Substances”, Bauman Moscow State Technical University, Moscow 105005, Russia; 3 Department of Testing Growth Regulators and Agrochemicals, Pryanishnikov All-Russian Scientific Research Institute of Agrochemistry, Moscow 127550, Russia; mtmasm@mail.ru; 4 Institute of Ecology, Biotechnology and Nature Management, Kazan Federal University, Kazan 420008, Russia; alesgordeev@kpfu.ru (A.S.G.); lrbiktasheva@kpfu.ru (L.R.B.); 5 Software Engineering, Institute of Computer Technologies and AI, Kyrgyz National University named after J. Balasagyn, Bishkek 720000, Kyrgyzstan; dmitry.mikhaylov@knu.kg; 6 Impactive Technologies Holding Limited, Abu Dhabi P.O. Box 95044, United Arab Emirates

**Keywords:** nanotechnology in agriculture, silver nanoparticles, apple fire blight, *Erwinia amylovora*, plant protection, phytopathogenic bacteria, exopolysaccharides, antioxidant enzymes, environmental impact, sustainable agriculture

## Abstract

The present study evaluated the phytoprotective activity of silver nanoparticles stabilized with sodium amphopolycarboxyglycinate (AgNPs-APCG) against apple fire blight caused by *Erwinia amylovora*. A highly colloidally stable AgNPs-APCG dispersion was prepared by borohydride chemical reduction and contained predominantly spherical metallic AgNPs with an average diameter of 10.4 ± 4.9 nm and a ζ-potential of −52.6 ± 2.4 mV. The obtained dispersion showed pronounced antibacterial activity against the *E. amylovora* strain, isolated from fire blight-affected shoots, with a MIC of 18.75 mg/L. In the field trial, foliar application at 5 and 25 mg/L reduced fire blight incidence and severity, with biological efficacies of 52.8% and 80.3%, respectively, and increased marketable yield by 12.0% and 16.7%. The phytoprotective efficacy of AgNPs-APCG at 5 mg/L, a concentration nominally below the in vitro MIC for *E. amylovora*, was consistent with a possible contribution of factors in addition to direct antibacterial activity. Supporting this possibility, AgNPs-APCG reduced carbohydrate-containing exopolysaccharide content in *E. amylovora* cultures to 51 ± 5% of the untreated control. Furthermore, higher activities of antioxidant enzymes were observed in AgNPs-APCG-treated apple leaves, while malondialdehyde content showed no pronounced increase, suggesting possible modulation of redox- and defense-related metabolism in treated plants. Potential environmental implications of the tested application regimes were considered based on the estimated Ag input to soil. These findings suggest that multiple pathogen- and host-associated responses may contribute to the phytoprotective effect of AgNPs-APCG and support its further evaluation as a multifunctional agent against bacterial plant diseases.

## 1. Introduction

Plant diseases caused by phytopathogenic microorganisms are among the major factors limiting crop productivity and the quality of agricultural products [1,2]. Among infectious plant diseases, those caused by phytopathogenic bacteria are of particular concern: current estimates attribute up to about 10% of global food-production losses to this group of infections [3,4]. Their impact becomes especially severe in high-value perennial fruit crops, where consequences of infection can compromise not only current-season yield and fruit quality but also the long-term productivity and survival of trees [5,6,7]. In apple production, the most serious bacterial threat is fire blight, a destructive disease caused by *Erwinia amylovora* (Burrill 1882), manifested by wilting and darkening of leaves, necrosis of flowers and young shoots, and browning, shriveling, or mummification of fruits [8].

A range of strategies is currently used to control diseases caused by phytopathogenic bacteria; however, each of these methods has its limitations [9,10]. Phytosanitary approaches, such as testing of planting material, equipment disinfection, crop rotation, spatial isolation, and microclimate management, can reduce pathogen introduction and spread but rarely provide complete protection under favorable infection conditions. The development and introduction of resistant cultivars require considerable time and financial investment, and their effectiveness may decrease as phytopathogens adapt to host resistance mechanisms [11]. Copper-based bactericides mainly act preventively on plant surfaces and have limited efficacy against developed infections inside plant tissues [12], whereas biological preparations often show variable field performance depending on environmental conditions and native microbiota [13]. The use of antibiotics is constrained by regulatory restrictions and the risk of development of bacterial drug resistance [14]. In addition, repeated application of the same control protocols may affect non-target microorganisms, including beneficial microbiota, and increase the chemical burden on agroecosystems. These limitations highlight the need for new phytoprotective strategies.

Several emerging approaches are being explored to overcome these limitations, including the use of bacteriophages [15], elicitors that activate nonspecific plant defense responses [16], quorum-sensing inhibitors and other strategies targeting bacterial cell-to-cell communication [17], as well as rapid pathogen identification based on omics technologies and digital monitoring tools [18], including artificial-intelligence-assisted systems [19]. In parallel, advances in nanotechnology have opened new opportunities for the development of plant-protection agents [20,21,22,23]. Silver nanoparticles (AgNPs) are of particular interest in this context because they may combine antimicrobial activity with the ability to modulate plant physiological responses [24,25,26,27]. AgNPs have repeatedly shown activity against phytopathogenic bacteria both in vitro and in planta, and their potential phytoprotective effect may arise from two complementary components: direct multitarget antibacterial action against pathogen cells and host-mediated responses associated with defense-related metabolism and induced resistance [28,29,30]. This combined mode of action may broaden the applicability of AgNPs-based preparations against different phytopathogenic bacteria and reduce the risk of rapid resistance development compared with single-target antibacterial compounds [31].

In terms of antibacterial activity, AgNPs act through both Ag^+^ release and nanoparticle-specific interactions with the cell envelope [27,32,33]. Released Ag^+^ ions bind to thiol and other electron-donor groups in membrane proteins and enzymes, disrupt electron and transmembrane transport, promote ROS formation, and can enter bacterial cells and interact with cytoplasmic components such as DNA and ribosomes, whereas adhesion of AgNPs to the bacterial surface may locally increase Ag^+^ concentration near the cell envelope and damage membrane structure [34,35,36,37]. Furthermore, AgNPs may affect traits directly involved in plant infection [38,39]. In phytopathogenic bacteria, AgNPs have been shown to affect chemotaxis, cell motility, biofilm formation, and exopolysaccharide production, which are involved in surface attachment, host colonization, and protection from plant defense responses [40,41,42]. Thus, AgNPs-based preparations may suppress plant bacterioses not only by killing pathogen cells but also by weakening colonization- and virulence-associated processes required for disease development [43,44,45].

In addition to pathogen-related effects, AgNPs may potentially contribute to disease suppression by modulating host plant defense responses [26,46,47]. After foliar or root exposure, AgNPs and released Ag^+^ can interact with plant tissues and trigger dose-dependent changes in redox homeostasis, antioxidant enzyme activity, photosynthetic performance, primary metabolism, and phytohormone-related pathways [48,49,50]. At moderate concentrations, AgNPs-induced ROS formation may function as a signaling process involved in oxidative burst, reinforcement of cell wall-associated barriers, phenolic metabolism, phytoalexin production, and systemic acquired resistance [51,52,53,54]. Proteomic studies also indicate that AgNPs exposure can affect proteins related to metal detoxification, cysteine/glutathione metabolism, biosynthesis of cell wall components, as well as chitinases and other pathogenesis-related proteins [55,56,57]. Therefore, under properly selected conditions, AgNPs may act as exogenous elicitor-like agents that activate plant defense-associated pathways and complement their direct antibacterial activity [25].

Despite the large number of studies addressing the antibacterial properties of AgNPs and the physiological consequences of their interaction with plants, most of them are focused on in vitro assays, seedling or young sprout models, or short-term tests on detached plant organs, whereas field studies on adult crop plants that combine bacterial plant disease suppression and the analysis of pathogen-related traits and host defense responses are scarce. Therefore, the aim of this study was to provide an integrated evaluation of the field efficacy, pathogen-associated effects, and host physiological responses to sodium amphopolycarboxyglycinate-stabilized silver nanoparticles (AgNPs-APCG), combining the assessment of fire blight suppression and apple yield in mature apple trees with investigations of antibacterial activity, carbohydrate-containing exopolysaccharide (EPS) content in *E. amylovora* cultures, and antioxidant enzyme activities and malondialdehyde content in apple leaves. The novelty of this work lies in the combined consideration of these complementary aspects within a single study, providing a more comprehensive understanding of the phytoprotective effects of AgNPs-APCG.

## 2. Results

### 2.1. Colloid-Chemical Characterization of AgNPs-APCG Dispersion

During the synthesis of the AgNPs-APCG dispersion, the addition of an AgNO_3_ solution to the APCG solution resulted in the appearance of a white turbidity in the reaction mixture, which was attributed to the formation of AgCl due to the interaction of AgNO_3_ with NaCl present in the commercial solution of APCG. After the subsequent addition of NaBH_4_, the color of the reaction mixture changed to yellow-brown, indicating the formation of AgNPs.

**Figure 1:**
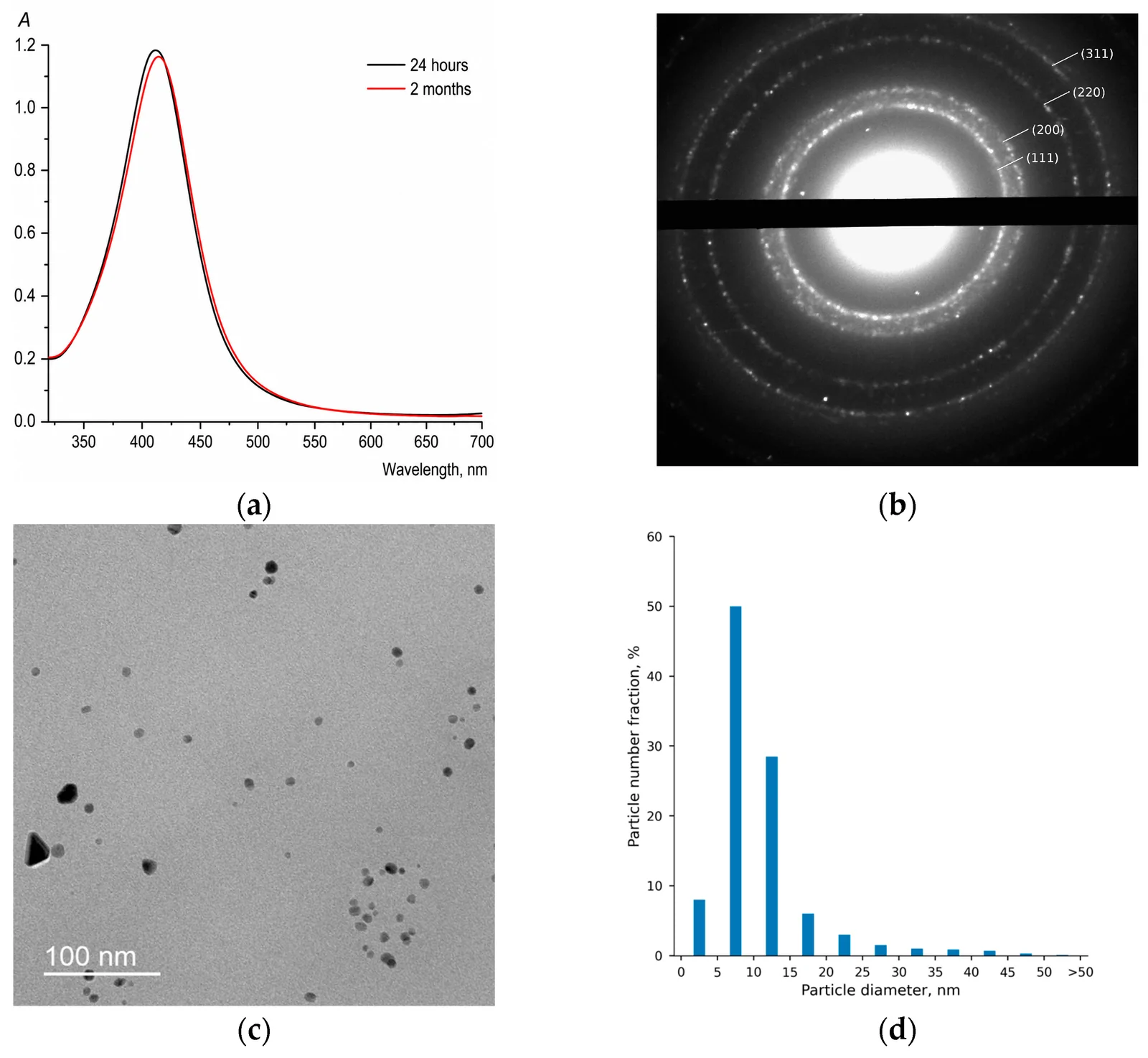
Colloid-chemical characterization of AgNPs-APCG dispersion: (**a**) UV–visible absorption spectra recorded 24 h after synthesis and after two months of storage; (**b**) SAED pattern of AgNPs-APCG; (**c**) TEM microphotograph; (**d**) particle size distribution determined from TEM images.

The UV–visible absorption spectrum of the obtained AgNPs-APCG dispersion contained a localized surface plasmon resonance (LSPR) band with a maximum at λ ≈ 410 nm, characteristic of AgNPs (Figure 1a); the intensity and position of this band remained almost unchanged during two months of storage. The measured interplanar spacings determined from the first four diffraction rings were 2.35, 2.02, 1.43, and 1.22 Å, corresponding to the (111), (200), (220), and (311) reflections, respectively, which corresponded to the standard values for metallic silver of 2.36, 2.04, 1.44, and 1.23 Å, respectively (Figure 1b) [58]. TEM analysis showed that AgNPs-APCG consisted mainly of nearly spherical NPs, along with individual faceted NPs, including truncated triangular NPs (Figure 1c); the particle size distribution was unimodal, and the average particle diameter was 10.4 ± 4.9 nm (Figure 1d). According to DLS measurements, the hydrodynamic diameter of AgNPs-APCG was 27.2 ± 5.3 nm, and the ζ-potential was −52.6 ± 2.4 mV.

### 2.2. Antibacterial Activity of AgNPs-APCG Against Test Bacterial Strains

The MIC values determined for AgNPs-APCG, AgNO_3_ and APCG without silver are presented in Table 1. AgNPs-APCG showed pronounced antibacterial activity against all tested bacterial strains. At 24 h after resazurin addition (48 h of total incubation), the MIC values of AgNPs-APCG were 6.25 mg/L for *P. aeruginosa* and 12.5 mg/L for both *E. coli* and *B. subtilis*. At 96 h after resazurin addition (120 h of total incubation), the MIC value remained unchanged for *P. aeruginosa* and *B. subtilis*, whereas for *E. coli* it increased to 25 mg/L. The antibacterial activity of AgNPs-APCG was comparable to that of AgNO_3_. In contrast, APCG without silver did not inhibit bacterial growth within the tested concentration range. The MIC determinations were fully reproducible across the four independent replicates, with identical results obtained for each substance–strain combination.

### 2.3. Activity of AgNPs-APCG Against Apple Fire Blight

#### 2.3.1. Detection of *E. amylovora* in Apple Shoots with Fire Blight Symptoms

Apple shoots collected from the experimental orchard showed typical symptoms of fire blight, including wilting and darkening of leaves, as well as browning or blackening of shoot tips. To confirm the presence of the causative agent, total DNA extracted from symptomatic shoot tissues was analyzed by real-time PCR using primers specific to *E. amylovora*. *E. amylovora* was detected in all five symptomatic shoot samples, with Ct values ranging from 24.2 to 31.4. The positive control containing *E. amylovora* DNA produced the expected amplification signal, whereas no amplification was detected in the no-template control. Melting-curve analysis revealed a single peak at approximately 80.8 °C, consistent with the melting behavior previously reported for the same primer pair by Jin et al. [59].

#### 2.3.2. Antibacterial Activity of AgNPs-APCG Against *E. amylovora*

**Figure 2:**
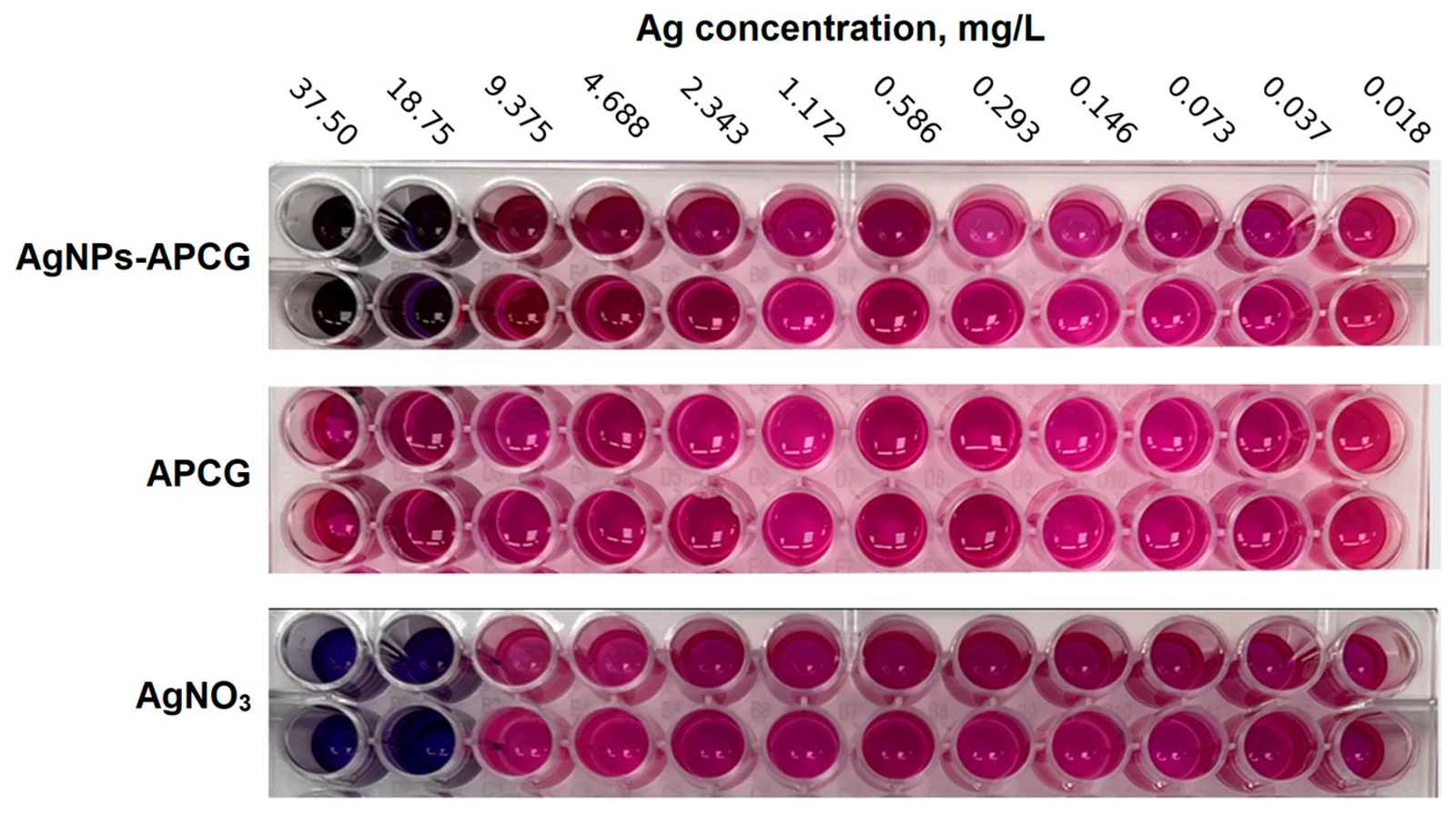
Representative image of a resazurin-based MIC assay for AgNPs-APCG, APCG without silver, and AgNO_3_ against *E. amylovora* recorded at 48 h of incubation. The APCG concentrations in the APCG without silver series were matched to those present in the corresponding AgNPs-APCG dilutions.

The antibacterial activity of AgNPs-APCG against phytopathogenic *E. amylovora* was evaluated in vitro by determining the MIC using a resazurin-based serial dilution assay. AgNO_3_ was used as an ionic silver reference, and APCG without silver was tested in parallel to assess the possible contribution of the stabilizer to antibacterial activity (Figure 2). AgNPs-APCG inhibited the growth of *E. amylovora* with a MIC = 18.75 mg/L at both 48 and 120 h of incubation. The same MIC value was determined for AgNO_3_. In contrast, APCG without silver did not inhibit the growth of *E. amylovora* within the tested concentration range. Identical MIC values were obtained in all four independent replicates for all tested substances.

#### 2.3.3. Efficacy of AgNPs-APCG Against Apple Fire Blight

AgNPs-APCG reduced the incidence and severity of apple fire blight under field conditions at both 5 and 25 mg/L tested concentrations (Table 2). In the untreated control, disease incidence on inflorescences at the flowering stage was 23.2%. Treatment with AgNPs-APCG reduced this value to 18.0% at 5 mg/L and to 12.4% at 25 mg/L.

A similar decrease in disease symptoms was observed on shoots at the shoot growth and fruit growth stages. Based on shoot disease severity at the fruit growth stage, the control efficacy of AgNPs-APCG was 52.8% at 5 mg/L and 80.3% at 25 mg/L.

#### 2.3.4. Effect of AgNPs-APCG on Carbohydrate-Containing EPS Content in *E. amylovora* Cultures

The relative carbohydrate-containing EPS content after treatment with AgNPs-APCG at ½ MIC (9.375 mg/L) was calculated from A_490_ values normalized to the optical density (OD_culture_) of the corresponding bacterial culture (Table 3). In the untreated control, the relative EPS content was taken as 100%. APCG without silver had no pronounced effect on this parameter, whereas silver-containing treatments decreased the relative EPS content: AgNO_3_ reduced it to 63 ± 5% of the untreated control, while AgNPs-APCG caused a stronger decrease, reducing the relative EPS content to 51 ± 5%, with a statistically significant difference between the two silver-containing treatments.

### 2.4. Effect of AgNPs-APCG Treatments on Marketable Yield

AgNPs-APCG treatments increased marketable yield compared with the untreated control (Table 4). The marketable yield in the control variant was 7.25 t/ha, whereas treatment with AgNPs-APCG at 5 and 25 mg/L increased it to 8.12 and 8.46 t/ha, respectively, corresponding to increases of 12.0% and 16.7%. Both increases exceeded the LSD_0_._05_ value, indicating a statistically significant effect.

### 2.5. Effect of AgNPs-APCG on Antioxidant Enzyme Activities and MDA Content in Apple Leaves

The activities of peroxidase (POD), catalase (CAT), and polyphenol oxidase (PPO), as well as malondialdehyde (MDA) content, were determined in apple leaves collected from untreated control trees and from the trees treated with AgNPs-APCG at 5 and 25 mg/L (Table 5). Relative to the untreated control, foliar treatment with AgNPs-APCG increased the activity of all three enzymes in a concentration-dependent manner. At 5 mg/L, POD, CAT, and PPO activities increased by 6%, 20%, and 22%, respectively, relative to the control. At 25 mg/L, the activities of POD, CAT, and PPO increased by 32%, 43%, and 34%, respectively. However, because the biochemical measurements were performed on technical replicates of pooled treatment-level samples, these differences are interpreted descriptively and were not subjected to inferential statistical comparison.

MDA content showed no pronounced increase after AgNPs-APCG treatment. In the untreated control, MDA content was 12.6 ± 3.5 nmol·g^−1^ FW, whereas the corresponding values at 5 and 25 mg/L were 14.1 ± 5.2 and 11.4 ± 2.8 nmol·g^−1^ FW, respectively; the mean ± SD intervals overlapped with the control interval for both treated variants.

## 3. Discussion

### 3.1. Colloid-Chemical Properties of AgNPs-APCG Dispersion

The obtained data demonstrate that APCG can effectively stabilize AgNPs formed by borohydride reduction in an aqueous medium. The presence of an LSPR band with a maximum near 410 nm is consistent with the formation of AgNPs, while the absence of pronounced spectral changes after two months of storage indicates that the dispersion retained the nanosized state and did not undergo pronounced aggregation; this is important for preserving the biological activity of AgNPs.

The TEM and SAED results show that the synthesized AgNPs were predominantly spherical with an average diameter of 10.4 ± 4.9 nm and a unimodal size distribution; however, individual faceted and truncated triangular nanoparticles were also observed. SAED analysis confirmed that the particles consisted of crystalline metallic silver.

The hydrodynamic diameter determined by DLS was higher than the particle diameter measured by TEM. This difference is explained by the fact that TEM measures the dried metallic core, whereas DLS measures particles in dispersion together with the adsorbed APCG layer and the associated hydration shell.

The highly negative ζ-potential of AgNPs-APCG (−52.6 ± 2.4 mV) indicates that electrostatic repulsion is the main mechanism contributing to the colloidal stabilization of the dispersion. The negative surface charge is most likely associated with the adsorption of APCG molecules on the AgNP surface and the presence of deprotonated carboxylate groups. Thus, APCG acted as a surface modifier that provided a negatively charged stabilizing layer around the AgNPs.

It should be noted that the distribution of silver between particulate and dissolved forms, including Ag^+^, was not quantified. Therefore, the biological effects discussed below cannot be attributed exclusively to particulate AgNPs, and a contribution from dissolved silver species cannot be excluded.

### 3.2. Antibacterial Activity of AgNPs-APCG Against Test Bacterial Strains

The MIC data demonstrate that AgNPs-APCG possess pronounced antibacterial activity against both Gram-negative and Gram-positive test bacterial strains (Table 1). The observed MIC values were in the range of 6.25–12.5 mg/L after 48 h of incubation and remained at the same level or increased only slightly after prolonged incubation for 120 h.

The activity of AgNPs-APCG was comparable to that of AgNO_3_, whereas APCG without silver did not inhibit bacterial growth within the tested concentration range. This shows that the antibacterial effect of the dispersion was determined by the silver-containing component rather than by the stabilizer itself. At the same time, APCG plays an important indirect role by stabilizing AgNPs in a nanosized state, which is necessary for maintaining their high surface area, colloidal stability, and consequently the ability to interact with bacterial cells.

### 3.3. Efficacy of AgNPs-APCG Against Apple Fire Blight

The presence of *E. amylovora* in symptomatic apple shoots was confirmed by real-time PCR, which supported its identification as the causal agent of the observed fire blight symptoms. The bacterium was isolated from symptomatic apple tissues, and the antibacterial activity of AgNPs-APCG against the isolated strain was then evaluated in vitro. The obtained MIC value was 18.75 mg/L, which is within the range of MIC values previously reported for AgNPs against this pathogen, typically varying from approximately 2 to 25 mg/L [43,60,61,62] depending on the experimental design and the nature of AgNPs used. Previous studies have attributed the antibacterial activity of AgNPs against *E. amylovora* mainly to interactions with the bacterial cell envelope, membrane damage, Ag^+^ release, ROS generation, leakage of intracellular components, and disruption of thiol-containing proteins and respiratory enzymes [63,64]. However, the MIC value obtained for *E. amylovora* exceeded the MIC values determined for most of the test bacterial strains (Table 1). This relatively high MIC may be associated with specific biological features of *E. amylovora*, including EPS-mediated formation of a protective extracellular polysaccharide layer around bacterial cells, biofilm-forming ability, the adaptation to oxidative stress encountered during host infection, etc. To evaluate field efficacy across different AgNPs-APCG concentrations, foliar applications at 5 and 25 mg/L were tested in the experimental orchard.

Under field conditions, AgNPs-APCG reduced the incidence and severity of apple fire blight in a concentration-dependent manner. The biological efficacy at 5 mg/L reached 52.8%, although this nominal concentration was below the MIC determined in vitro for the isolated *E. amylovora* strain. This observation suggests that mechanisms other than direct growth inhibition may also contribute to protection in planta including host-mediated responses. Increasing the Ag concentration to 25 mg/L increased the biological efficacy to 80.3%; the stronger effect at this concentration may reflect a greater contribution of direct antibacterial activity of AgNPs-APCG. However, the nominal concentration of the applied dispersion does not directly represent the local concentration or time-dependent exposure of bacteria. For example, spray drying and retention may affect the local concentration of AgNPs, whereas repeated applications, nanoparticle persistence, Ag^+^ release, and other factors may alter the duration and chemical form of silver exposure. Moreover, the MIC represents the concentration required to completely inhibit visible bacterial growth under broth conditions, whereas concentrations below the MIC may still partially suppress bacterial growth. Consequently, the nominal foliar concentration and the in vitro MIC cannot be directly compared quantitatively, and assessment of the relative contributions of direct antibacterial and host-mediated mechanisms requires further investigation.

The reduction in disease severity was accompanied by an increase in marketable yield. Foliar treatment with AgNPs-APCG increased marketable yield by 12.0% at 5 mg/L and by 16.7% at 25 mg/L relative to the untreated control, indicating that application of the dispersion not only suppressed fire blight development but also provided an agronomically relevant increase in fruit productivity.

Because the field trial included only an untreated control and AgNPs-APCG treatments, the obtained data demonstrate the efficacy of the complete formulation but do not allow the separate contribution of APCG or a field-specific advantage of nanoparticulate over ionic silver to be established. In addition, no conventional fire blight treatment was included as a reference; therefore, the observed efficacy cannot be directly compared with current standard control practices.

### 3.4. Biological Responses Associated with AgNPs-APCG Treatment

The phytoprotective effect of AgNPs-APCG against apple fire blight observed in the field trial at both nominal sub-MIC and above-MICs suggests that it may involve mechanisms in addition to direct antibacterial activity. Such mechanisms may include modulation of host redox- and defense-related responses, and, potentially, suppression of virulence-associated bacterial traits. To assess these possible mechanisms, the present study evaluated carbohydrate-containing EPS content in *E. amylovora* cultures under subinhibitory conditions, as well as antioxidant enzyme activities and MDA content in apple leaves.

#### 3.4.1. Effect of AgNPs-APCG on Carbohydrate-Containing EPS Content

Extracellular polymeric substances are secreted macromolecular components that form an extracellular matrix around bacterial cells and play a key role in their adhesion to plant surfaces and host tissues, cell aggregation, biofilm formation, and protection of bacterial cells from environmental stresses, antimicrobial agents, and host defense responses [40]. For phytopathogenic bacteria, including *E. amylovora*, EPS production is therefore directly relevant to host colonization and infection development. AgNPs-APCG reduced the relative carbohydrate-containing EPS content in *E. amylovora* cultures under subinhibitory concentrations to 51 ± 5% of the untreated control. Since the assay was performed at ½ MIC and A_490_ values were normalized to OD_culture_, the decrease indicates lower carbohydrate-containing EPS content per unit of bacterial culture density rather than a simple reduction in bacterial growth. This suggests that AgNPs-APCG may interfere with bacterial metabolic processes involved in EPS production and accumulation even at concentrations that do not completely inhibit bacterial growth. The observed decrease in carbohydrate-containing EPS content may affect the extracellular protective polymeric layer of *E. amylovora*, potentially reducing biofilm-associated persistence and increasing the susceptibility of bacterial cells to environmental stresses and host defense responses [65]. However, because the assay determines the total carbohydrate content of the isolated extracellular polymeric material, this decrease cannot be attributed specifically to individual virulence-associated polysaccharides such as amylovoran or levan. Notably, AgNO_3_ reduced this value to 63 ± 5%, indicating a less pronounced effect than that observed for AgNPs-APCG. This difference may suggest an additional contribution of the nanoparticulate form of silver, consistent with the AgNP–cell envelope interaction discussed in the literature [26]. At the same time, APCG without silver had no pronounced effect, indicating that the observed decrease in EPS content was associated with the silver-containing component rather than with the stabilizer itself. It should also be noted that the EPS content assay was performed at ½ MIC (9.375 mg/L), rather than at the 5 mg/L concentration used in the field trial; therefore, the laboratory observation cannot be directly extrapolated to the response observed in the field.

#### 3.4.2. Effect of AgNPs-APCG on Antioxidant Enzyme Activities and Lipid Peroxidation

Another component of the phytoprotective effect of AgNPs-APCG may be associated with changes in the pro-/antioxidant balance in plant tissues. Reactive oxygen species (ROS), on the one hand, are potentially damaging molecules capable of inducing oxidative stress and lipid peroxidation; on the other hand, at relatively low concentrations, they function as key secondary messengers in redox-dependent signaling pathways involved in plant adaptation and defense. Such ROS-dependent signaling may contribute to reinforcement of cell wall-associated barriers, oxidation of phenolic substrates, production of phytoalexins and other antimicrobial secondary metabolites, and systemic resistance responses [51,52,53,54]. Therefore, moderate changes in antioxidant enzymatic activities may indicate the induction of adaptive responses rather than the development of oxidative damage.

Foliar treatment with AgNPs-APCG was associated with higher activities of POD, CAT, and PPO in a concentration-dependent manner. POD and PPO are involved in phenolic metabolism, oxidative cross-linking reactions, and cell wall-associated defense responses [66,67], whereas CAT contributes to the regulation of H_2_O_2_ levels and maintenance of redox homeostasis [68,69]. Thus, the observed changes in the enzymatic profile are consistent with changes in redox regulation related to plant defense in treated plants. At the same time, these changes may reflect both the response to AgNPs-APCG exposure and differences in infection status among the treatments. Importantly, this enzymatic response was not accompanied by a consistent concentration-dependent increase in MDA content, a marker of lipid peroxidation, at the sampling time. Taken together, these observations may be consistent with a regulated antioxidant and defense-associated response that may potentially contribute to host-mediated protection against fire blight.

Partly consistent with these observations, treatment of *E. amylovora*-infected pear trees with sesame-oil-derived AgNPs was previously shown to lower the levels of oxidative stress markers, including MDA and H_2_O_2_, and to improve fruit set and yield [70].

### 3.5. Environmental and Food-Safety Considerations

To assess the practical environmental and food-safety implications of the tested AgNPs-APCG treatments, the amount of silver applied per tree and the corresponding theoretical input to soil were estimated.

At an application volume of 1100 L/ha, AgNPs-APCG concentrations of 5 and 25 mg/L corresponded to 5.5 and 27.5 g Ag/ha per application, respectively, or cumulative seasonal doses of 22 and 110 g Ag/ha after four applications. Based on an application volume of approximately 2 L per tree, these treatments corresponded to 10 and 50 mg Ag per tree per application and 40 and 200 mg Ag per tree over the season, respectively.

To estimate the potential accumulation of silver in soil, a conservative upper-bound estimate was calculated in which the entire applied Ag dose was assumed to reach the soil and remain within the upper 0.10 m layer, with no losses through surface runoff, leaching, plant uptake, or other pathways. The theoretical increase in soil Ag concentration was therefore calculated as:

(1) $$C_{\mathrm{soil}}=\frac{D_{Ag}}{m_{\mathrm{soil}}}=\frac{\omega_{Ag}\times V_{\mathrm{dispersion}}\times k\times n}{A\times h\times \rho_{\mathrm{soil}}}=\frac{25\times 1100\times 4\times 10}{10,000\times 0.1\times 1300}=0.846\;\mathrm{mg}/\mathrm{kg}$$

where C*_soil_* is the theoretical silver concentration in soil, *D_Ag_* is the cumulative amount of Ag applied per hectare (mg/ha), *m_soil_* is the mass of the considered soil layer (kg/ha), *ω*_A*g*_ = 25 mg/L is the Ag concentration in the working dispersion, *V*_dispersion_ = 1100 L/ha is the application volume, *k* = 4 is the number of applications per year, n = 10 is the number of years of application, *A* = 10,000 m^2^ is the treated area, *h* = 0.1 m is the considered soil depth, and *ρ*_soil_ = 1300 kg/m^3^ is the soil bulk density.

For context, regulatory and regulatory-derived soil benchmarks for silver reported by different jurisdictions generally range from several to tens of milligrams per kilogram of dry soil [71,72,73]. Under the conservative assumptions used here, annual application of AgNPs-APCG over 10 years would result in theoretical treatment-derived increases in soil Ag concentrations of 0.169 and 0.846 mg/kg for the 5 and 25 mg/L treatments, respectively; thus, even the highest estimated increase would remain well below these soil-quality and ecological screening benchmarks.

Nevertheless, the environmental fate of Ag after foliar application is complex and may involve retention on plant surfaces, transfer to soil, chemical transformation, leaching, and interactions with soil biota. Potential effects on non-target organisms therefore require further investigation. In addition, because Ag residues in soil and harvested fruit were not directly determined in the present study, direct measurements are required to establish the long-term environmental and food-safety profile of repeated AgNPs-APCG application.

## 4. Materials and Methods

### 4.1. Synthesis of AgNP Dispersion

**Figure 3:**
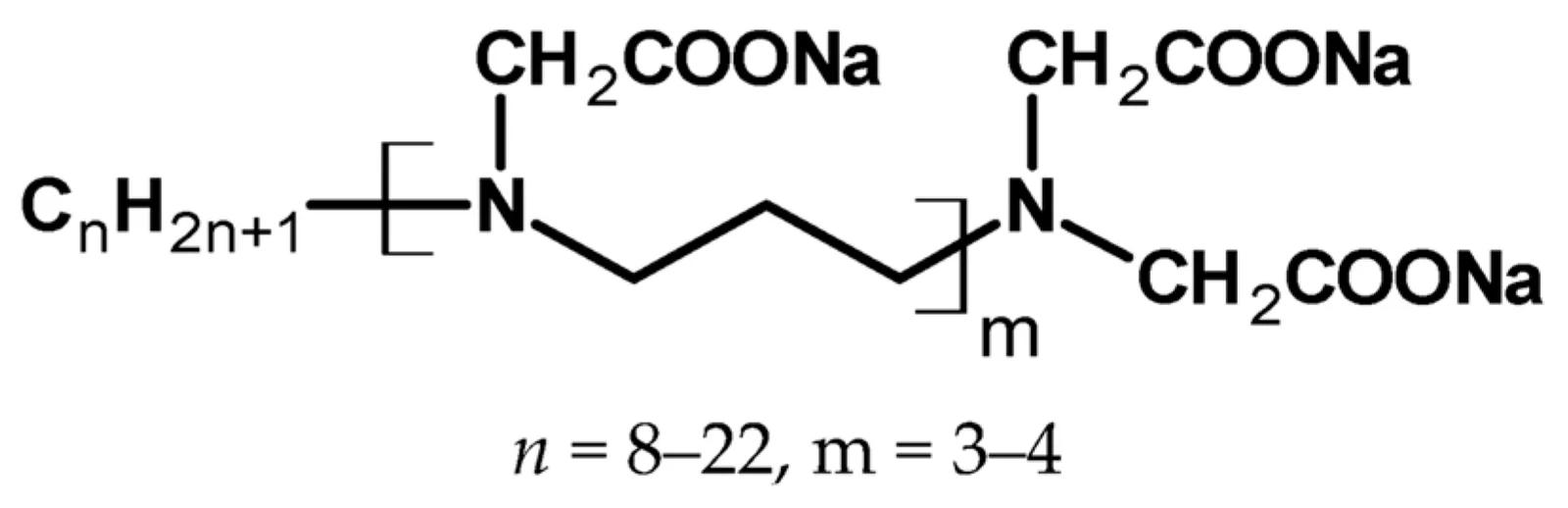
Structural formula of APCG.

Silver nanoparticle dispersion stabilized with sodium APCG (30 wt.%, Akzo Nobel, Amsterdam, The Netherlands), the structural formula of which is given in Figure 3, was prepared by chemical reduction of silver nitrate (99%, Ruskhim, Moscow, Russia) with sodium borohydride (99%, Reaktiv, Moscow, Russia) in aqueous medium [74,75]. Distilled water was used in all synthesis procedures.

To obtain 1 L of AgNPs-APCG dispersion, an aqueous solution containing 2.00 g of active APCG, corresponding to a Ag mass ratio of 1:8, was first prepared in 500 mL of distilled water. Then, 250 mL of an aqueous AgNO_3_ solution containing 0.3937 g of AgNO_3_ was added dropwise to the APCG solution under continuous stirring. After that, 250 mL of an aqueous NaBH_4_ solution containing 0.1761 g of NaBH_4_ was added dropwise as a reducing agent. The formation of AgNPs was visually indicated by the appearance of a yellow-brown coloration of the reaction mixture. After complete addition of the reducing agent, the dispersion was stirred for an additional 2 h until hydrogen evolution ceased. The resulting AgNPs-APCG dispersion with a nominal silver concentration of 250 mg/L calculated from the amount of AgNO_3_ added was used as a stock dispersion and diluted with distilled water to the required working concentrations before subsequent experiments.

### 4.2. Characterization of AgNP Dispersions

#### 4.2.1. UV-Visible Spectroscopy

UV–visible absorption spectra of AgNPs-APCG were recorded using a UV-1800 spectrophotometer (Shimadzu, Kyoto, Japan) in the wavelength range of 330–700 nm. Measurements were performed in quartz cuvettes with an optical path length of 10 mm. Before analysis, the 250 mg/L AgNPs-APCG dispersion was diluted with distilled water to a silver concentration of 10 mg/L.

#### 4.2.2. TEM and SAED

The morphology and size distribution of AgNPs were examined by transmission electron microscopy (TEM), and their crystalline structure was analyzed by selected area electron diffraction (SAED), using a JEM-2100 microscope (JEOL, Tokyo, Japan) operated at an accelerating voltage of 200 kV. Samples were prepared by placing 1–2 μL of an AgNPs-APCG dispersion with a silver concentration of 100 mg/L onto carbon-coated copper grids, followed by drying in air at room temperature. The average particle diameter was calculated from measurements of at least 250 individual particles. SAED patterns were recorded using the same instrument, and interplanar spacings were determined from the diffraction-ring diameters using the microscope diffraction calibration. The diffraction rings were indexed by comparison with the reference interplanar spacings of face-centered cubic metallic silver [58].

#### 4.2.3. Dynamic Light Scattering

The hydrodynamic diameter and ζ-potential of AgNPs-APCG were determined by dynamic light scattering (DLS) method using a Zetasizer Nano ZS90 analyzer (Malvern Instruments, Malvern, UK). Measurements were performed in quartz cuvettes with an optical path length of 10 mm at a silver concentration of 50 mg/L and at room temperature. The scattering angle was 90°. ζ-Potential measurements were performed using a dip cell electrode immersed in the sample. The obtained data were processed using DynALS v2.0 software with a monomodal Gaussian distribution model. The reported hydrodynamic diameter and ζ-potential values are presented as mean ± standard deviation (SD) based on at least four independent measurements.

#### 4.2.4. Antibacterial Activity

The antibacterial activity of AgNPs-APCG was evaluated by determining the minimum inhibitory concentration (MIC) using a serial dilution method with a resazurin-based viability assay. The assay was performed against *Pseudomonas aeruginosa* ATCC 27853, *Bacillus subtilis* 168, *Escherichia coli* ATCC 25922 and *Erwinia amylovora* VRN-23. The bacterial strains were cultivated in LB medium containing 10 g/L NaCl, 10 g/L peptone, and 5 g/L yeast extract. Precultures of *P. aeruginosa*, *B. subtilis*, and *E. coli* were incubated at 37 °C for 12 h, whereas the preculture of *E. amylovora* was incubated at 28 °C for 12 h. The resulting bacterial precultures were standardized to approximately 3.5 × 10^7^ CFU/mL based on OD_600_ measurements using a previously established calibration curve relating optical density to viable bacterial cell concentration (CFU/mL). The bacterial inoculum was prepared by adding 600 μL of the standardized preculture to 6 mL of fresh LB medium.

The assay was performed in sterile 96-well microplates. A volume of 100 μL of the bacterial inoculum was added to each well. AgNPs-APCG, AgNO_3_, and APCG solution without silver were added to the first well of each dilution series, followed by two-fold serial dilutions. Each assay well contained 100 μL of bacterial inoculum and 100 μL of the corresponding test-compound dilution, resulting in a total assay volume of 200 μL and a final bacterial concentration of approximately 1.5 × 10^6^ CFU/mL. For the assays with *P. aeruginosa*, *B. subtilis*, and *E. coli*, the highest Ag concentration in the test-compound solution was 100 mg/L, corresponding to 50 mg/L after 1:1 mixing with the bacterial inoculum. For *E. amylovora*, the dilution series was prepared from 75 mg/L Ag, corresponding to a maximum final concentration of 37.5 mg/L in the assay wells. APCG without silver was tested at concentrations corresponding to those present in the respective AgNPs-APCG dispersions. All Ag concentrations reported for AgNPs-APCG and AgNO_3_ are expressed on an elemental silver basis.

After inoculation and addition of the tested compounds, the microplates containing *P. aeruginosa*, *B. subtilis*, and *E. coli* were incubated at 37 °C for 24 h, whereas the microplates containing *E. amylovora* VRN-23 were incubated at 28 °C for 24 h. Following incubation, bacterial growth was visually assessed based on culture turbidity before the addition of resazurin. Subsequently, 10 μL of a 0.1% (*w*/*v*) aqueous resazurin solution was added as a bacterial viability indicator. The resazurin response was evaluated visually at 24 and 96 h after indicator addition. A color change from violet to pink was considered indicative of bacterial metabolic activity. The MIC was defined as the lowest concentration of the tested agent that inhibited visible bacterial growth and did not produce a resazurin color change indicative of bacterial metabolic activity.

Each assay included a bacterial growth control containing bacterial inoculum and LB medium without the tested antimicrobial agent and a sterility control containing sterile LB medium without bacterial inoculum. Cell-free controls containing AgNPs-APCG, AgNO_3_, or APCG in LB medium without bacterial cells were used to evaluate possible direct effects of the tested compounds on the resazurin indicator. A cell-free LB medium/resazurin control without test compounds was additionally included as an indicator control. Possible interference of the tested compounds with the resazurin readout was assessed by comparing the corresponding cell-free compound controls with the indicator control. In addition, bacterial growth was visually assessed based on culture turbidity before resazurin addition, providing confirmation of bacterial growth or inhibition. All experiments were performed in four independent replicates.

### 4.3. Detection of E. amylovora in Symptomatic Apple Shoots

The detection of *E. amylovora* in symptomatic apple shoots was performed by real-time PCR following the general diagnostic framework recommended by EPPO diagnostic protocol PM 7/20(3) [8]. Shoot samples showing typical symptoms of fire blight, including leaf wilting and darkening as well as browning or blackening of the shoot tip, were collected from five affected apple trees. One symptomatic shoot sample was taken from each tree. The samples were placed in polyethylene bags with filter paper to prevent condensation and transported to the laboratory for analysis.

Total genomic DNA was extracted from the border region between visually healthy and affected shoot tissues using a FastDNA SPIN Kit for Soil (Bio101, Qbiogene, Heidelberg, Germany) according to the manufacturer’s instructions. Real-time PCR was performed using the primer pair RS19160_201F (5′-CTG TTT CAA ATT TGG CGA TGT A-3′) and RS19160_201R (5′-AAG GTG TTT TGT GCT GTT TGT AT-3′), previously described for the detection of *E. amylovora* [59]. Amplification was carried out on a CFX96 Touch real-time PCR detection system (Bio-Rad, Munich, Germany).

Each 25 μL reaction mixture contained 1× SYBR Green PCR buffer, 2.5 mM MgCl_2_, 200 μM of each dNTP, 0.2 μM of each primer, 0.2 μL of SynTaq DNA polymerase (5 U/μL), and 1 μL of template DNA. The amplification protocol consisted of an initial denaturation step at 95 °C for 15 min, followed by 39 cycles of denaturation at 95 °C for 30 s, primer annealing at 60 °C for 30 s, and extension at 72 °C for 30 s.

After amplification, melting curve analysis was performed to verify the specificity of the PCR products. Each PCR run included a no-template control and a positive control containing DNA of *E. amylovora*.

### 4.4. Isolation of E. amylovora from Symptomatic Apple Shoots

An *E. amylovora* isolate used in subsequent in vitro assays was obtained from symptomatic apple shoots following the culture-based isolation procedure described in EPPO PM 7/20(3) [8]. Briefly, tissue fragments excised from the border region between visually healthy and affected shoot tissues were surface-disinfected with 70% ethanol, rinsed twice in sterile distilled water, macerated in sterile phosphate-buffered saline, and the resulting extract, along with its ten-fold dilutions, was spread onto nutrient sucrose agar and incubated at 26 °C for 72 h. Single mucoid levan-positive colonies typical of *E. amylovora* were subcultured to purity, and the identity of the resulting isolate (designated *E. amylovora* VRN-23) was confirmed by real-time PCR with the RS19160_201F/RS19160_201R primer pair performed on DNA extracted from the pure culture (as described in Section 4.3); this isolate was used in all MIC and EPS assays and was maintained in LB medium supplemented with 20% (*v*/*v*) glycerol at −80 °C.

### 4.5. Field Trials

The field trial was conducted during the 2024 growing season in an experimental apple orchard located in the Paninsky District of the Voronezh Region, Russia. The experiment was performed under natural orchard conditions without artificial inoculation or environmental control on 8-year-old apple trees (*Malus domestica* Borkh.) of the cultivar ‘Rossoshanskoe Polosatoe’, planted at a spacing of 6.0 × 3.0 m. The orchard had a documented history of fire blight: during the preceding 2023 growing season, shoot disease incidence at the fruit growth stage was 14.1%, with a mean disease severity of 12.5%.

Standard orchard management practices included autumn sanitary pruning, grassing of inter-row spacing, and four irrigation events per season. Each experimental plot included five trees and was considered an independent field replicate, with four replicates established for each treatment. The experimental plots were arranged within the existing orchard layout and randomly assigned to the three treatment groups, with four independent replicate plots per treatment; adjacent plots were separated by one untreated tree serving as a buffer. Each tree was sprayed individually with precautions taken to minimize spray drift to adjacent plots. The experiment included the following treatments:

1. Untreated control;
2. Four foliar spray applications of AgNPs-APCG at a silver concentration of 5 mg/L;
3. Four foliar spray applications of AgNPs-APCG at a silver concentration of 25 mg/L.

Treatments were applied at the green cone, early bloom, fruit set, and fruit growth stages. The application rate of the working dispersion was 1100 L/ha, corresponding to approximately 2 L per tree. No other fungicides or bactericides were applied to the experimental trees during the field trial.

The incidence of fire blight symptoms on inflorescences was assessed during flowering on 100 inflorescences per tree (selected from each of four main scaffold branches oriented toward the four cardinal directions), corresponding to 500 inflorescences per experimental plot, and calculated as the percentage of affected inflorescences showing typical symptoms, including darkening, wilting, and blight-like necrosis of flowers or pedicels, relative to the total number of assessed inflorescences. The incidence and severity of fire blight symptoms on shoots were assessed twice: during the period of active shoot growth after flowering and during the fruit growth stage. At each assessment, 40 shoots per tree were evaluated, corresponding to 200 shoots per experimental plot. Ten shoots were selected from each of four main scaffold branches oriented toward the four cardinal directions. Shoot disease incidence (*P*, %) was calculated as:

(2) P=nN×100%

where *n* is the number of affected shoots and *N* is the total number of assessed shoots.

Disease severity or disease development (*R*, %) was calculated based on the proportion of the affected shoot length:

(3) R=∑biN

where *b_i_* is the percentage of affected length of the *i*-th shoot and *N* is the total number of assessed shoots.

The biological efficacy (*E*, %) of AgNPs-APCG against apple fire blight was calculated as:

(4) $$E=\frac{R_{\mathrm{control}}-R_{\mathrm{treatment}}}{R_{\mathrm{control}}}\times 100\%$$

where *R_control_* is disease severity in the untreated control and *R_treatment_* is disease severity in the AgNPs-APCG-treated variants.

At harvest, marketable apples (excluding those with visible disease symptoms, mechanical damage, or other defects affecting marketability) were weighed separately for each experimental plot, and the resulting marketable yield was recalculated per hectare. Yield increase was calculated as the difference between the marketable yield of AgNPs-APCG-treated trees and that of the untreated control.

### 4.6. Determination of Extracellular Carbohydrate-Containing Polymers Content

The effect of AgNPs-APCG on the content of extracellular carbohydrate-containing polymeric substances (carbohydrate-containing EPS) in cultures of *E. amylovora* was evaluated under subinhibitory conditions. For this purpose, a bacterial suspension of *E. amylovora* was incubated in LB medium for 24 h at 28 °C with orbital shaking at 150 rpm with AgNPs-APCG at a concentration of 9.375 mg/L, corresponding to ½ MIC (18.75 mg/L). AgNO_3_ at its ½ MIC was used as an ionic silver reference, while an APCG solution without silver was tested at the concentration corresponding to that present in the AgNPs-APCG treatment. Untreated bacterial culture was used as a control. Four independent bacterial cultures were prepared and processed separately for each treatment and were considered independent experimental replicates.

After incubation, the OD_culture_ was measured at 600 nm, with dilution in sterile medium when necessary. Cell-free controls containing LB medium and the corresponding concentrations of AgNPs-APCG, AgNO_3_, or APCG were used to correct for formulation-related background at OD_600_; a medium-only blank containing LB without bacteria or test compounds was also included. Bacterial cells were then removed by centrifugation at 6000× g for 10 min at 4 °C, and the resulting cell-free supernatant was used for isolation of the extracellular polymeric fraction. One milliliter of the supernatant was mixed with 3 mL of 96% ethanol pre-cooled to −20 °C and incubated for 12–16 h at 4 °C. The precipitated extracellular polymeric fraction was collected by centrifugation at 10,000× g for 10 min, washed with 70% ethanol, air-dried, resuspended in 1 mL of distilled water, and incubated at 37 °C for 30 min with intermittent mixing. Carbohydrate content in the isolated extracellular polymeric fraction was determined spectrophotometrically using the phenol–sulfuric acid method according to DuBois et al. [76]. A 200 μL aliquot of the resuspended fraction was mixed with 200 μL of 5% phenol and 1 mL of concentrated sulfuric acid. After color development for 30 min at room temperature, the samples were centrifuged at 10,000× *g* for 5 min, and absorbance was measured at 490 nm. Matrix controls containing LB medium and the corresponding concentrations of AgNPs-APCG, AgNO_3_, or APCG without bacteria, as well as an LB medium blank without bacteria or test compounds, were subjected to the same sample-preparation procedure. The A_490_ values were corrected for the corresponding matrix background and normalized to the blank-corrected ODculture to account for differences in bacterial culture density between treatments.

Relative EPS content was calculated as the ratio of the mean normalized EPS value for each treatment to that of the untreated control and expressed as a percentage. The uncertainty of the relative EPS content was calculated by propagation of the standard deviations of the corresponding treatment and control normalized EPS values.

### 4.7. Antioxidant Enzyme Activities in Apple Leaves

Leaf samples for enzymatic analysis were collected 48 h after the fourth and final foliar application, during the fruit growth stage, from trees in the untreated control and the 5 and 25 mg/L AgNPs-APCG treatment groups. Fully expanded mature leaves were collected from current-year shoots, with five leaves sampled from each of four main scaffold branches oriented toward the four cardinal directions on each of the five trees in each of the four field plots per treatment. Leaf material collected from all trees and field plots within each treatment was pooled to obtain a composite sample. Immediately after sampling, plant material was frozen in liquid nitrogen and stored at −80 °C until analysis. Before analysis, the frozen composite leaf material was thoroughly ground and mixed, and four 200 mg subsamples representing technical replicates were taken. Each subsample was homogenized in 2 mL of ice-cold 50 mM phosphate buffer (pH 6.8–7.2) containing 1 wt.% polyvinylpyrrolidone. The homogenate was centrifuged at 12,000× *g* for 15 min at 4 °C, and the resulting supernatant was used for the determination of antioxidant enzyme activities. For all enzyme assays, activities were normalized to tissue fresh weight using the extract volume (2 mL), assay aliquot volume (0.1 mL), and sample fresh mass (0.2 g), corresponding to a normalization factor of 100.

#### 4.7.1. Peroxidase (POD)

POD activity was determined spectrophotometrically using the guaiacol method according to Chance and Maehly [77]. The reaction mixture contained 1.7 mL of 50 mM phosphate buffer (pH 7.0), 200 μL of 0.1 M guaiacol, 100 μL of 0.15% hydrogen peroxide, and 100 μL of enzyme extract. The increase in absorbance at 470 nm (A_470_) was recorded every 15 s for 3 min. One unit (U) of POD activity was defined as an increase in A_470_ of 0.01 per minute; activity was calculated from the linear rate of absorbance change (ΔA_470_/min), applying the normalization factor described above, and expressed as U·g^−1^ FW.

#### 4.7.2. Catalase (CAT)

CAT activity was determined according to Aebi [78] by monitoring the decomposition of hydrogen peroxide. The reaction mixture contained 1.9 mL of 10 mM H_2_O_2_ prepared in 50 mM phosphate buffer (pH 7.0) and 100 μL of enzyme extract. The decrease in absorbance at 240 nm was recorded every 10 s for 1 min. CAT activity was calculated from the initial rate of decrease in absorbance at 240 nm using the molar extinction coefficient of H_2_O_2_ (ε_240_ = 39.4 M^−1^·cm^−1^), accounting for the reaction volume (2 mL) and the normalization factor described above. Activity was expressed as µmol H_2_O_2_ decomposed·min^−1^·g^−1^ FW.

#### 4.7.3. Polyphenol Oxidase (PPO)

PPO activity was determined using catechol as a substrate according to the assay principles described by Mayer et al. [79]. The reaction mixture contained 1.8 mL of 50 mM phosphate buffer (pH 6.8), 100 μL of 0.33 M catechol, and 100 μL of enzyme extract. The increase in absorbance at 420 nm was recorded every 10 s for 3 min. One unit (U) of PPO activity was defined as an increase in absorbance at 420 nm of 0.01 per minute under the assay conditions. Activity was calculated from the linear rate of absorbance change (ΔA_420_/min), applying the normalization factor described above, and expressed as U·g^−1^ FW.

### 4.8. Determination of MDA Content

Malondialdehyde (MDA) content in apple leaf tissues was determined using a simplified thiobarbituric acid-reactive substances (TBARS) method based on [80] without additional corrections for TBA-independent absorbance and carbohydrate-related interference. Frozen apple leaf samples (200 mg) were homogenized in 2 mL of 5% trichloroacetic acid (TCA) on ice using a pre-cooled mortar and pestle.

The homogenate was centrifuged at 12,000× *g* for 15 min at 4 °C, and the resulting supernatant was used for analysis. An aliquot of the supernatant was mixed with 0.5% thiobarbituric acid (TBA) prepared in 20% TCA. The reaction mixture was heated at 95 °C for 30 min and then rapidly cooled in an ice bath to stop the reaction. After cooling, the mixture was centrifuged again to remove precipitated material.

The absorbance of the supernatant was measured at 532 nm, and nonspecific absorbance was corrected by subtracting the absorbance at 600 nm. MDA concentration was calculated using an extinction coefficient of 155 mM^−1^ cm^−1^ for the MDA–TBA complex. The results were expressed as nmol MDA per g fresh weight.

### 4.9. Statistical Analysis

Quantitative data are presented as mean ± standard deviation (SD). Field trial data and EPS assay data were analyzed by one-way analysis of variance (ANOVA). Disease incidence and severity were analyzed separately for each phenological stage. Normality and homogeneity of variance were assessed using the Shapiro–Wilk and Levene tests, respectively. Fisher’s least significant difference (LSD) test was used for pairwise comparisons at the 5% significance level (*p* < 0.05). Leaf biochemical data based on technical replicates of pooled samples are presented descriptively as mean ± SD without inferential statistical comparisons.

## 5. Conclusions

In this study, an aqueous dispersion of silver nanoparticles stabilized with sodium amphopolycarboxyglycinate (AgNPs-APCG) was obtained and evaluated for its efficacy in suppressing apple fire blight. The dispersion was characterized with high colloidal stability, contained predominantly spherical metallic AgNPs with an average TEM diameter of 10.4 ± 4.9 nm, along with a small fraction of faceted triangular particles and had a ζ-potential of −52.6 ± 2.4 mV. Furthermore, the AgNPs-APCG dispersion exhibited pronounced antibacterial activity against the test strains *P. aeruginosa*, *E. coli*, and *B. subtilis*, with MIC values of 6.25–25 mg/L.

The MIC value of AgNPs-APCG against fire blight causal phytopathogen *E. amylovora* was determined to be 18.75 mg/L; concentrations of 5 and 25 mg/L were selected for the field trial to evaluate phytoprotective efficacy across nominal sub-MIC and above-MIC conditions. Under field conditions, disease incidence on inflorescences decreased from 23.2% in the untreated control to 18.0% and 12.4% after treatment with 5 and 25 mg/L AgNPs-APCG, respectively; biological efficacy on shoots reached 52.8% and 80.3%. Of particular practical importance, the disease suppression was accompanied by increased marketable yield, from 7.25 t/ha in the untreated control to 8.12 and 8.46 t/ha at 5 and 25 mg/L, respectively, corresponding to increases of 12.0% and 16.7%.

The phytoprotective activity of AgNPs-APCG observed at 5 mg/L, nominally below the in vitro MIC determined for *E. amylovora*, suggests that, in addition to direct antibacterial activity, other pathogen- and host-associated responses may contribute to fire blight suppression in planta. These observations are consistent with a possible combined contribution of direct antibacterial activity and additional pathogen- and host-associated responses. AgNPs-APCG reduced the relative carbohydrate-containing EPS content in *E. amylovora* cultures to 51 ± 5% of the untreated control, suggesting that the dispersion may affect EPS biosynthesis, secretion, or accumulation. Since EPSs are involved in bacterial attachment to plant surfaces, biofilm matrix formation, cell protection, and host tissue colonization, this effect may be relevant to colonization- and virulence-associated functions of the extracellular matrix and therefore may attenuate the pathogen’s virulence potential. Furthermore, higher activities of the antioxidant enzymes POD, CAT, and PPO were observed in AgNPs-APCG-treated apple leaves. At the same time, this enzymatic response was not accompanied by a consistent concentration-dependent increase in MDA content. These observations may reflect a redox-related adaptive response in plant tissues that may contribute to fire blight suppression.

Overall, the obtained results show that the phytoprotective effect of AgNPs-APCG is accompanied by direct antibacterial activity, reduced EPS content, and changes in redox-related responses in host tissues. These findings support further consideration of AgNPs as a promising multifunctional agent for the control of bacterial plant diseases. Further studies should focus on elucidating the molecular mechanisms of AgNP-mediated disease suppression, including effects on bacterial virulence and host defense signaling pathways, the relative contributions of AgNPs and released Ag^+^ to the observed biological effects, residual silver levels in plant tissues and soil, environmental fate and transformation of silver species, non-target effects, and efficacy under different agroecological and epidemiological conditions.

## Figures and Tables

**Table 1:** Minimum inhibitory concentrations of AgNPs-APCG, AgNO_3_, and APCG against test bacterial strains.

Tested Substance	Incubation Time, h	*P. aeruginosa*, mg/L	*E. coli*, mg/L	*B. subtilis*, mg/L
AgNPs-APCG	48	6.25	12.5	12.5
	120	6.25	25	12.5
AgNO_3_	48	3.125	6.25	12.5
	120	12.5	12.5	12.5
APCG	48	>400	>400	>400
	120	>400	>400	>400

**Table 2:** Efficacy of AgNPs-APCG against apple fire blight caused by *E. amylovora*.

Treatment №	Ag Concentration, mg/L	Inflorescences	Shoots				Efficacy to Control, %
		Flowering Stage	Shoot Growth Stage		Fruit Growth Stage		
		Disease Incidence, %	Disease Incidence, %	Disease Severity, %	Disease Incidence, %	Disease Severity, %	
1	0	23.2 ± 3.2	17.3 ± 2.0	10.8 ± 1.6	19.5 ± 2.2	12.7 ± 1.7	–
2	5	18.0 ± 2.8	9.1 ± 1.8	5.4 ± 1.4	9.3 ± 1.9	6.0 ± 1.5	52.8
3	25	12.4 ± 2.3	6.3 ± 1.4	3.8 ± 1.1	6.3 ± 1.6	2.5 ± 1.2	80.3
LSD_0.05_	–	4.4	2.8	2.2	3.1	2.4	–
Treatment *p*-value	–	0.0012	<0.001	<0.001	<0.001	<0.001	-

**Table 3:** Relative carbohydrate-containing EPS content in *E. amylovora* cultures after treatment with AgNPs-APCG and reference compounds under subinhibitory conditions.

Tested Substance	A_490_	OD_culture_	Normalized EPS Value, A_490_/OD_culture_	Relative EPS Content, % of Control
Control	0.401 ± 0.018	0.823 ± 0.030	0.487 ± 0.028	–
APCG *	0.387 ± 0.019	0.811 ± 0.028	0.477 ± 0.029	98 ± 8
AgNO_3_	0.242 ± 0.014	0.789 ± 0.025	0.307 ± 0.020	63 ± 5
AgNPs-APCG	0.193 ± 0.012	0.776 ± 0.025	0.249 ± 0.017	51 ± 5
LSD_0.05_	0.025	0.042	0.037	–
*p*-value	<0.001	0.111	<0.001	–

* APCG was tested at 75 mg/L, which corresponded to its concentration in the AgNPs-APCG treatment.

**Table 4:** Effect of AgNPs-APCG treatments on marketable yield.

Treatment №	Ag Concentration, mg/L	Marketable Yield, t/ha	Yield Increase, t/ha	Yield Increase, %
1	0	7.25 ± 0.22	–	–
2	5	8.12 ± 0.20	0.87	12.0
3	25	8.46 ± 0.18	1.21	16.7
LSD_0.05_	–	0.32	–	–
*p*-value	–	<0.001	–	–

**Table 5:** Antioxidant enzyme activities and MDA content in apple leaves after treatment with AgNPs-APCG.

Treatment №	Ag Concentration, mg/L	POD Activity (U·g^−1^ FW)	CAT Activity (µmol H_2_O_2_·g^−1^ FW·min^−1^)	PPO Activity (U·g^−1^ FW)	MDA Content (nmol·g^−1^ FW)
1	0	1046 ± 112	132 ± 15	52.1 ± 5.9	12.6 ± 3.5
2	5	1110 ± 126	159 ± 18	63.4 ± 7.1	14.1 ± 5.2
3	25	1378 ± 149	188 ± 21	69.8 ± 7.9	11.4 ± 2.8

## Data Availability

The original contributions presented in this study are included in the article and Supplementary Materials. Further inquiries can be directed to the corresponding authors.

## Supplementary Materials

The following supporting information can be downloaded at: https://www.mdpi.com/article/10.3390/plants15203208/s1. File S1: Replicate-level experimental data and calculations used to prepare Table 2, Table 3, Table 4 and Table 5.

## Abbreviations

The following abbreviations are used in this manuscript:

AgNPs	Silver nanoparticles
AgNPs-APCG	Silver nanoparticles stabilized with sodium amphopolycarboxyglycinate
ANOVA	Analysis of variance
APCG	Sodium amphopolycarboxyglycinate
CAT	Catalase
CFU	Colony-forming units
DLS	Dynamic light scattering
EPPO	European and Mediterranean Plant Protection Organization
EPS	Extracellular polymeric substances
FW	Fresh weight
LB	Luria–Bertani medium
LSD	Least significant difference
LSPR	Localized surface plasmon resonance
MDA	Malondialdehyde
MIC	Minimum inhibitory concentration
NPs	Nanoparticles
OD_culture_	Optical density of the corresponding bacterial culture
PCR	Polymerase chain reaction
POD	Peroxidase
PPO	Polyphenol oxidase
ROS	Reactive oxygen species
SAED	Selected area electron diffraction
SD	Standard deviation
TBA	Thiobarbituric acid
TBARS	Thiobarbituric acid-reactive substances
TCA	Trichloroacetic acid
TEM	Transmission electron microscopy
